# Anti-tumor necrosis factor patent expiration and the risks of biocopies in clinical practice

**DOI:** 10.1186/s13075-014-0501-5

**Published:** 2014-12-06

**Authors:** Morton Scheinberg, Gilberto Castañeda-Hernández

**Affiliations:** Clinical Research Center Hospital AACD and Hospital Albert Einstein, Av Albert Einstein 627, São Paulo, 05652000 Brazil; Department of Pharmacology, Cinvestav, Av. Instituto Politécnico Nacional 2508, Mexico, 07360 DF Mexico

## Abstract

Biosimilars that were not compared in clinical trials with the compound innovator are not true biosimilars (biocopies) and are associated with risks that the clinical rheumatologist should be aware of before generalized use. This article comments on various aspects surrounding the use of such biocopies in clinical rheumatology.

The introduction of targeted biologic therapies has revolutionized the treatment of immune inflammatory arthritis [1]. The use of such compounds has significantly improved patient outcomes and decreased the need for surgical procedures to repair damaged joints [2]. The use of tumor necrosis factor (TNF) antagonists has proven successful since their introduction in 1999. The worldwide sales of etanercept, infliximab, and adalimumab have reached over $20 billion (USD), and these medications are constantly in the top-ten list of blockbuster drugs [3]. Some rheumatologists advocate that for patients with less severe disease they be used earlier than more traditional therapies for rheumatoid arthritis. However, pricing has been a major drawback for such implementation because of the negative impact on public health-care costs. In Brazil, patients who failed treatment with conventional therapy and have active disease have access to more expensive medications to control their arthritis [4]. The Ministry of Health spends over $600 million (USD) yearly to provide access to biologics and this amount accounts for over 50% of the available budget for free medications to public health patients with chronic diseases. However, there is great enthusiasm for patent expirations with the expectation that the introduction of biosimilars will substantially reduce the burden to the public health system, a similar reduction having occurred with the use of biosimilars for erythropoietin [5-7].

The European Medicine Agency (EMA) has already approved the first biosimilar version of infliximab from Celltrion (Incheon, Korea) and Hospira (Lake Forest, IL, USA) for rheumatoid arthritis, paving the road for less expensive medications when they finally reach the market in 2015. Also important was the EMA recommendation for approval of the infliximab biosimilar for ankylosing spondylitis, psoriatic arthritis, psoriasis, and, surprisingly, inflammatory bowel disease, in which the pathogenetic role of TNF is still not fully understood [8-11]. Whether gastroenterologists will be comfortable with this ‘bridging’ strategy remains to be seen. Outside of the European Union and the US, the regulatory framework for biosimilars varies considerably. Some countries adopted the World Health Organization (WHO) guidelines, which are very similar to those of the EMA. Canada has its own guidelines that present minor differences with those of the US Food and Drug Administration, but no approved biosimilar has been approved in the US.

In some countries in Latin America, non-innovator biologics have been approved by using legislation in place for synthetic copies of brand compounds (that is, without comparative clinical studies with the innovator). Therefore, these products cannot be considered biosimilars; rather, they are biocopies, also known as intended copies or non-regulated biologics [12,13]. The first was Etanar in Colombia; Etanar is a biocopy of etanercept manufactured in China and licensed as a new biologic. The second products were biocopies of rituximab. Reditux, a biocopy manufactured by the Indian company Dr. Reddy’s (Hyderabad, India), is licensed in Ecuador, Peru, Chile, Bolivia, and Paraguay. In Mexico, the local manufacturer, Probiomed (Mexico City), commercialized a biocopy of rituximab under the brand name Kikuzabam. In March 2014, owing to several adverse events, this biocopy was withdrawn from the market and its registration was nullified. More recently, in Mexico, two biopsies of etanercept were introduced in the market. One is the Chinese product which in Mexico has the brand name Etart. The other one is Infinitam, produced by Probiomed. In Mexico, there is already legislation that requires comparability trials with the reference biologic, but the request for marketing these products was introduced before the new regulation was effective. Surprisingly, the Mexican biocopies have been purchased at prices similar to those of the innovator products [14]. Therefore, savings for the social security system are minimal or non-existent. It appears that what led the Mexican regulatory agency to cancel the registration of the biocopy of rituximab was the frequent occurrence of anaphylactic reactions when switching between the two commercialized rituximab products [15]. At present, there is automatic substitution of etanercept and rituximab innovators and biocopies at the pharmacy level, without the physician being informed which product is actually dispensed. Moreover, Mexican biocopies may be used in the US, as American citizens frequently purchase their medication in Mexico because of its cheaper prices [16]. From the above, the lack of a uniform approach to the regulatory approval of true biosimilars versus biocopies (intended copies) in Latin America is clear.

In Brazil, the risk of registration of biocopies is null since the current legislation is similar to the recommendations from the WHO and the EMA. The Brazilian national agency (ANVISA) requests that a complete pre-analytical dossier and a rigorous comparison with the reference product be available. To stimulate the advent of biosimilars, the Ministry of Health is sponsoring an initiative known as Productive Partnership for Development, which will lead to domestic production of biosimilars through a transfer of technology agreement with international companies that are developing production of biosimilars, such as Sandoz (part of Novartis, Basel, Switzerland), Samsung (Seoul, Korea), Samsung Bioepis (Incheon, Korea), and Epirus Biopharmaceuticals, Inc. (Boston, MA, USA). Merck Serono (Darmstadt, Germany) has recently signed a partnership with a new Brazilian company (Bionovis, Barueri, Brazil) to ensure transfer of technology for the production of biosimilars during the next 5 years. The rituximab product from Merck Serono has yet to produce data of comparability in arthritis, a reason for concern since the products available at present are biocopies instead of true biosimilars. Orygen (Parkville, Australia), another biotechnology company, has signed a production partnership with Pfizer Inc. (New York, NY, USA) for technology transfer of adalimumab, infliximab, and rituximab.

In Europe, only biosimilars are expected to be approved and the risks of biocopies are avoided. How rheumatologists will deal with the fact that approval of a biosimilar was extended to all indications of Remicade (Janssen, Horsham, PA, USA) although Celltrion and Hospira undertook phase 1 and 3 studies in only rheumatoid arthritis and ankylosing spondylitis remains to be seen.

The use of biocopies does not lead to the necessary safety that is associated with the prescription of the innovator or a true biosimilar when they become licensed for use in clinical practice. Owing to the absence of rigorous clinical testing, biocopies should not be expected to have almost identical efficacy. Another issue that can generate confusion in clinical practice is the heterogeneity present in various countries in post-marketing surveillance programs by the health agencies when biocopies are exchanged with the innovator in an uncontrolled manner. In addition, nomenclature terminology regarding the use of the innovator versus biocopies is still an open issue [6,7]. The lack of mechanisms to rapidly identify toxicity issues, we believe, relates to the delay in the withdrawal of the biocopy of rituximab in Mexico.

Finally, the gradual acceptance of the first true biosimilar by rheumatologists will depend on real-life exposure. Norwegian authorities plan to address this issue this year by funding a head-to-head study of Inflectra (Hospira) (a biosimilar) versus Remicade in a bid to convince rheumatologists to prescribe the biosimilar because of its similarities in efficacy and safety and cost savings. The stimulation of switching back and forth by the Norwegian health authorities has yet to be adopted by any other country in Europe, and how this interchangeability approach will develop in the near future remains to be seen [17].

## Abbreviations

EMA: European Medicine Agency
TNF: tumor necrosis factor
WHO: World Health Organization
